# Tumors1Read at the May meeting of the Keystone Veterinary Medical Association.

**Published:** 1901-11

**Authors:** J. M. Carter

**Affiliations:** Philadelphia, Pa.


### TUMORS.[1]

By J. M. Carter, V.M.D.,
PHILADELPHIA, PA.

At our last meeting the subject of tumors was brought up, and in the discussion that followed it seemed that some of us had forgotten our pathology relative to this subject, and when the Secretary asked me to prepare something for this meeting I thought it might not be time wholly lost to review this subject of tumors, both as to their pathology and origin and as we find them in practice.

A tumor in the broadest sense of the word is an abnormal swelling in any part of the body. This definition would include all abscesses and cysts and all inflammatory processes, while in an ordinary sense we mean by a tumor those growths which appear without any apparent cause and continue to grow indefinitely, possessing an atypical structure and not exercising any function of service to the body.

Many causes and theories for these growths have been advanced from time to time. Some of these theories seem to have little evidence to bear them out, while others seem to be very applicable and plausible in certain forms. Virchow advanced the theory of

Read at the May meeting of the Keystone Veterinary Medical Association.

external irritation, which, in cases of carcinoma of the mammary gland and epithelioma of the lip of man, it would seem plausible; but how often with the same causes no abnormal growths are stimulated. Cohnheim advanced the theory that these growths were due to misplaced cells of the original blastoderm, which at some subsequent time have started to proliferate and develop. There seem to be certain forms which might be thus explained, as the teretoma or dermoid cysts and some of the benign encapsulated tumors. Other theories have been advanced. Some writers have tried to explain these growths by a retrograde change in the vital properties of certain cells, so that they tend to develop toward the original mesoblastic cells, and in an indefinite manner; but these theories seem to be purely speculative. Recently an infectious character has been ascribed to certain forms, especially the malignant tumors and those giving metastasis, and, where the general health is so much impaired, they do seem to resemble other infectious processes. Several forms of protozoa have been found and described in certain carcinomas and believed to be the possible and probable cause of the growth, but nothing definite has been proven. Whatever may be eventually proven to be the active and primary causes of the different abnormal growths, certain predisposing causes are often of great importance. Anything that devitalizes the tissues at the seat of the growth, whether it be from pressure, thermic, mechanical, or chemical irritation, or from general debility following disease or old age, may facilitate the growth, providing the active agent is present, whether it be a misplaced cell or a microörganism.

As to the histological structure of tumors in general, we might say that they do not differ absolutely from healthy tissue, but in nearly all cases they conform more or less to the normal tissue from which they grow. The cells may be larger or smaller than normal, or resemble the embryonal or undeveloped tissue, but any definite arrangement of the cells seems to be entirely wanting. We might also say that the character of a tumor is always closely related to the tissue from which it springs—that is to say, a connective-tissue growth always comes from tissue containing connective-tissue cells, and epithelial growths from tissue containing epithelial cells. Although tumors are often very closely connected with the surrounding tissue and of the same character, they add nothing whatever to the development or strength of the part, but are entirely parasitical, living wholly at the expense of the other tissues.

### *The Different Forms in Detail.*

I have secured from the museum of the Veterinary Department of the University of Pennsylvania some specimens of the different forms of tumors, which we will look over and examine a little more in detail.

*Fibroma*, or a connective-tissue tumor. These tumors may be hard or soft, depending upon the character of the intercellular substance. Fibromas are generally found as round, encapsulated tumors. The most common seats of origin are the subcutaneous connective tissue, the submucous tissue of the nose, the intermuscular connective tissue, the uterus, nerves, and mammary glands. In the uterus it almost always contains muscle tissue or a myofibroma. In the mammary gland it is often lobulated. On section of fibroma, especially hard fibroma, the fibres are arranged in circles, and have a white, glistening appearance; mucoid degeneration is common, making the tumor softer and showing a jelly-like appearance. Fatty degeneration is common, giving the section a yellowish color. Calcareous deposits are also frequently seen.

*Myxoma* is a benign connective-tissue tumor with an intercellular substance largely of mucoid material. These are soft, more or less flabby growths, enclosed in a capsule and having a rounded outline, or may be polypoid. The most common seats of origin are the mucous membrane and mammary gland. On section the substance shows a gelatinous appearance, resembling the vitreous humor of the eye. Very often other forms of tumors undergo the mucoid change and show this appearance; in fact, pure myxomas are seldom seen in the domestic animals.

*Lipoma* is a benign connective-tissue tumor, usually circumscribed and encapsulated, often having a lobulated character. These tumors, although of slow growth, often reach enormous size. On section they show the appearance of fatty tissue, but mostly more fibrous than normal fatty tissue.

Lipoma is common in the domestic animals, and always shows the appearance of the normal fat of the animal from which it comes. In cows it is most common in the subcutaneous tissue of the back and shoulders, and is whiter. In the dog, frequently about the synovial folds of joints and tendons, and more fibrous than lipoma of the horse. In the horse it is most common in the subserous tissue of the peritoneum, where it is often pendulous, and may become detached and lie free in the abdominal cavity. Lipoma is also occasionally found in the mammary gland and kidney.

*Chrondoma* is a tumor composed largely or entirely of cartilage.

*Ecchondroma*, or cartilage tumor proper, must be distinguished from ecchondrosis or cartilaginous outgrowths from bone from irritation, particularly where fractures have occurred and in rheumatoid arthritis. True chondromata are hard, resisting, mostly multiple tumors of slow growth, usually small, but may reach considerable size. They mostly take their origin from bone periosteum or true cartilage, but are occasionally met with in glandular organs, especially the parotid gland. The structure is usually hyaline when coming from bone or cartilage, and fibrous when in glandular structure. Calcification is very common. In dogs, chondroma is mostly seen in the mammary gland, while in the horse along the ribs, and multiple. In the human family, in the hands and feet.

*Osteoma* is a tumor composed of osseous material, and as in chondroma we must distinguish the exostosis or inflammatory outgrowths due to injury or some irritation from true bone tumors. The exostoses are direct outgrowths and of a more or less warty nature, while true osteoma are more extensive and present more the appearance of bony deposit, and are less closely attached. The character or structure of the two deposits is very similar, and may be either hard or spongy bone. Osteomata are of slow growth, and usually spring from bone or cartilage, but occasionally from serous membrane. Most common seats are about the epiphyses of long bones and maxillary bones, especially of the horse. When from serous membrane, mostly from the dura mater of the falx cerebri.

*Lymphangioma*, or lymphatic tumor. It is hard to separate these from dilatation of lymphatic vessels due to obstruction and causing hyperplastic processes as seen in elephantiasis of the extremities, while true lymphatic tumors are rarely if ever seen in the domestic animals. In the human family lymphangioma of the tongue, called macroglossia, and of the lip, macrocheilia, are occasionally seen as congenital tumors.

*Angioma.* A tumor-like formation composed principally of bloodvessels. Strictly speaking, these are seldom true tumors, and rarely show proliferation, but simply a dilatation and elongation of existing bloodvessels. The arteries and capillaries are mostly involved. They rarely project above the surface, and only show a red or port-wine colored spot on depigmented skin. The skin is the most common seat, where they are most always congenital as birthmarks. They are also seen in the liver as cavernous angioma.

*Lymphadenomata* are a class of tumors very closely resembling sarcoma. They are very malignant, give metastasis, and are confined to the lymphatic glands. They closely resemble the lymphatic enlargements from specific infections, as tuberculosis, but these are not true tumors, though in some of the domestic animals, especially ruminants, in the uterus and true stomach, there is a well-circumscribed true lymphatic tumor occasionally found.

*Sarcoma* is a very malignant tumor, composed of connective-tissue cells with very little intercellular substance. The connective-tissue cells of sarcoma are not perfectly developed, but resemble granulation tissue or embryonal tissue in various stages of development, do not tend to form perfect connective tissue, but to proliferate and reproduce its own kind. Sarcomata are more or less rounded tumors, frequently encapsulated, but of the primary tumors they are often irregular and infiltrating. The secondary tumors are nodular and nearly always encapsulated. They are of a fleshy consistency, thus the name sarcoma, which means fleshy, and mostly of a pink color on section, but may be white or grayish. Microscopically, sarcomata are divided according to the stage of the development of the connective tissue ; these may be small or large round cells or small or large spindle cells, or giant cells. Sarcomatous tissue is often mixed with other connective-tissue tumors, especially fibroma; also myxoma, osteoma, and adenoma. The malignancy of sarcoma depends upon the development of the cell composing it, the small, round-celled forms and melanotic or pigmented forms being the most malignant. Metastasis takes place entirely through the bloodvessels, as the lymphatics of the vicinity are not affected. To distinguish these forms macroscopically, we find the spindle-celled forms are of a pink color on section and are more dense and fibrous in appearance than the round-celled forms, and mostly come from dense connective tissue, as the periosteum, tendons, and fascia. These are the most common form and generally benign. The round-celled forms spring from loose connective tissue, as the subcutaneous and glandular connective tissue. They are much softer and of a milky-white appearance.

*Melanotic sarcoma* is any pigmented sarcoma, but especially the small, round-celled forms. These are found wherever pigment normally occurs—the skin, the eye, and pia mater. The giant-celled sarcoma is the most common form in bone. They are of slow growth and firmly attached to the bone structure.

*Endothelioma*, a tumor of purely endothelial origin, is found only on serous membrane. It closely resembles cancer. Has never been seen in domestic animals.

*Glioma.* A tumor made up of neuroglia or the connective tissue of the brain. It has never been found in domestic animals.

*Neuroma* is a term applied to a tumor made up of nerve fibres. These are extremely rare, and some authorities claim never exist, and that the tumor is simply a fibroma on the nerve, or, more often, the proliferative perineurium and endoneurium from irritation or amputation of the nerve, seen after neurotomies or amputation of a limb.

*Leiomyoma* is a tumor containing non-striated muscular fibres. They are usually connected with fibroma as benign encapsulated tumors.

*Rhobdomyoma* is a tumor containing striped muscular tissue, always found with some other tumor, mostly sarcoma ; they are likely congenital, and an example of misplaced cell.

*Papilloma.* A tumor arising from the surface of the integument or mucous membrane, and closely resembling the structure of the normal papillæ of the skin. The most familiar form is the wart and cutaneous corns. They are usually hard when occurring on the skin, and soft when on the mucous membrane. They are benign, as their tendency is to grow out and often disappear spontaneously.

*Adenoma* is a term applied to a new growth corresponding in structure to certain epithelial glands forming acini and tubules. They are usually encapsulated, benign tumors, and likely congenital. The most common seat is about the pylorus and duodenal glands. In the dog, especially of the rectum, affecting the anal glands and the thyroid gland or true goitre.

*Carcinomata,* or true cancer, are epithelial growths atypically reproducing certain glandular or other structures and showing a manifest tendency to irregular extension. The traumatic and parasitic theory, together with old age and general impaired health, seem to be the most likely causes for these growths. I might say here that Prof. Gaylord, in a recent article in a medical journal, claims to have isolated a protozoa, with which he has produced cancer in a large number of cases, and feels very sure he has found the true cause for certain cancers. Of course, this will have to be taken up by other investigators and verified before it becomes an established fact.

Carcinomata differ much in appearance in different parts of the body. In the skin they are usually more or less flat elevations with nodular surface and very frequent ulcerations. When coming from loose mucous membrane they are mostly somewhat polypoid with nodular ulcerative surface. When occupying glandular structure they are large, nodular tumors, with irregular infiltration and extension into the surrounding tissue. In consistency these tumors may be very hard or quite soft from degenerative changes. On section they are of a white or grayish appearance and very frequently a milky exudate from the surface. Primary cancers are rarely encapsulated, but show deep penetration into the surrounding tissue, while secondary cancers are frequently encapsulated. Primary cancers are usually of slow growth, but may be rapid, as in acute carcinosis. Secondary cancers are always more rapid than primary. In the horse squamous cancer of the skin and external genitals are the most frequent seats. In the dog the mammary gland, the uterus, and stomach are the most frequent seats. Cancer of the mammary gland is often multiple, many nodules, and gives metastasis to the lymphatics, liver, and marrow of bones. The microscopical appearance of cancer varies with the structure from which it springs, which it always resembles to a certain extent. The cells are arranged in acini and tubules, breaking through the limiting membrane and proliferating and spreading in all directions, with generally dense connective-tissue stroma. Degenerative changes are common, especially fatty and cystic formations, in mammary cancer, and horny formation in squamous cancer of the skin. The ulcerative surface of cancer is mostly inflammatory, from irritation and infection of the pus germs. Carcinomata are very malignant, and tend to recur after removal, and give metastasis, especially through the lymphatics and occasionally through the veins, to the liver and spleen. The general health of a patient suffering from cancer is affected very materially, likely due to a toxæmia, or it may be due to interference with some organic function when affecting the stomach or liver. There are several varieties of cancer classified by their histological formation, as epithelioma of the skin or mucous membrane, formed mostly of squamous epithelium, and showing more the arrangement of the papillæ of the skin, but taking a reverse direction. Glandular cancers are those showing alveolar or medullary arrangement.

*Teratoma* or *dermoid cysts* might be mentioned as a form of tumor, but might better be considered as monstrosities. These are always congenital formations, and seem to show some separate fœtal disposition as to their origin. They are most frequently found in the ovary, but have occurred elsewhere. Their inner surface resembles the skin, and may contain hair; sebaceous glands and teeth have been found.

As to the frequency of tumors in domestic animals, Fröhner gives the following statistics:

From 86,113 animals treated at the Berlin, Munich, and Dresden Veterinary Hospitals, 1131, or 1.3 per cent., were affected with tumors.

From 85,537 dogs treated at the same hospitals, 4020, or 4.7 per cent., were for tumors.

From 4972 bovines, 102, or 2 per cent., were for tumors; and horses, 1.3 per cent. for tumors.

From 643 dogs treated at Berlin for tumors, 262, or 40 per cent., were for carcinoma; 97, or 13 per cent., for fibroma; 65, or 10 per cent., for papilloma; 44, or 7 per cent., for sarcoma; 39, or 6 per cent., for lipoma; 2, or 0.3 per cent., for angioma.

From 47 horses treated for tumors, 10, or 21 per cent., were for sarcoma; 17, or 36 per cent., for papilloma; 6, or 13 per cent., for fibroma; 3, or 6 per cent., for carcinoma; 1, or 2 per cent., for lipoma; 1, or 2 per cent., for osteoma.

From 75 bovines affected with tumors, 20, or 27 per cent., were for sarcoma; 2, or 2.7 per cent., for carcinoma.

These tables are very incomplete, and cover a very small number of cases, but the percentage gives an approximate idea as to their respective frequency in the different species.

